# Near-Ultrasonic Transfer Function and SNR of Differential MEMS Microphones Suitable for Photoacoustics

**DOI:** 10.3390/s23052774

**Published:** 2023-03-03

**Authors:** Judith Falkhofen, Marcus Wolff

**Affiliations:** 1 Heinrich Blasius Institute of Physical Technologies, Hamburg University of Applied Sciences, 20099 Hamburg, Germany; 2 School of Computing, Engineering and Physical Sciences, University of the West of Scotland, Scotland High Street, Paisley PA1 2BE, UK

**Keywords:** photoacoustic spectroscopy, MEMS microphones, ultrasound, deconvolution, SNR, sensitivity

## Abstract

Can ordinary *Micro-Electro-Mechanical-Systems* (MEMS) microphones be used for near-ultrasonic applications? Manufacturers often provide little information about the signal-to-noise ratio (SNR) in the ultrasound (US) range and, if they do, the data are often determined in a manufacturer-specific manner and are generally not comparable. Here, four different air-based microphones from three different manufacturers are compared with respect to their transfer functions and noise floor. The deconvolution of an exponential sweep and a traditional calculation of the SNR are used. The equipment and methods used are specified, which makes it easy to repeat or expand the investigation. The SNR of MEMS microphones in the near US range is mainly affected by resonance effects. These can be matched for applications with low-level signals and background noise such that the highest possible SNR can be achieved. Two MEMS microphones from Knowles performed best for the frequency range from 20 to 70 kHz; above 70 kHz, an Infineon model delivered the best performance.

## 1. Introduction

In photoacoustic spectroscopy, energy is applied to a sample by means of modulated laser excitation. The absorbed energy is converted into a local temperature increase, which results in expansion of the gas, followed by relaxation [1]. This pressure or sound wave is usually inaudible due to small amplitude and, often, high frequency. Therefore, photoacoustic methods require very sensitive sound sensors.

Micro-Electro-Mechanical-Systems (MEMS) microphones have many advantages in the application, as they are produced cheaply and, due to their compact form, can be integrated in small sensors. Many MEMS microphones can be applied in the ultrasonic range, even if they have not been explicitly designed for this purpose. However, above the first mechanical resonance, the sensitivity of the microphone is strongly frequency dependent [2]. Therefore, the analysis of the transfer function is particularly relevant. In addition, the signal-to-noise ratio (SNR) is often quantified by the manufacturers only up to 20 kHz. Therefore, a thorough characterization is advisable before selecting a microphone. MEMS microphones have been the subject of reviews [3,4] and are considered as ultrasonic transducers, especially for photoacoustic imaging [5].

Photoacoustic sensors often take advantage of resonators to amplify the acoustic signal. Since the PA signal is inversely proportional to the volume of the cell (or the resonator), often high frequencies in the ultrasound range occur. For instance, the resonant frequency of the optimized resonator for non-invasive glucose measurement falls within the near-ultrasound (US) range [6,7]. Frequencies between 20 kHz and 1 MHz are also of interest for PA imaging, as these relatively low frequencies come along with less acoustic attenuation. Therefore, deeper tissue layers can be investigated [8,9].

Here we compare transfer functions and SNRs of four differential MEMS microphones in the frequency range between 20 and 80 kHz. Section 2 presents the experimental setup and the applied methods. Section 3 provides the results followed by a conclusion.

## 2. Materials and Methods

### 2.1. Materials

After a systematic market study, the four MEMS microphones listed in Table 1 were selected for the comparative investigation. The sensitivity and the SNR, both measured by the manufacturer with a sinewave of 1 kHz and 94 dB SPL and noise from 20 Hz to 20 kHz, were extracted from the datasheet.

**Figure 1:**
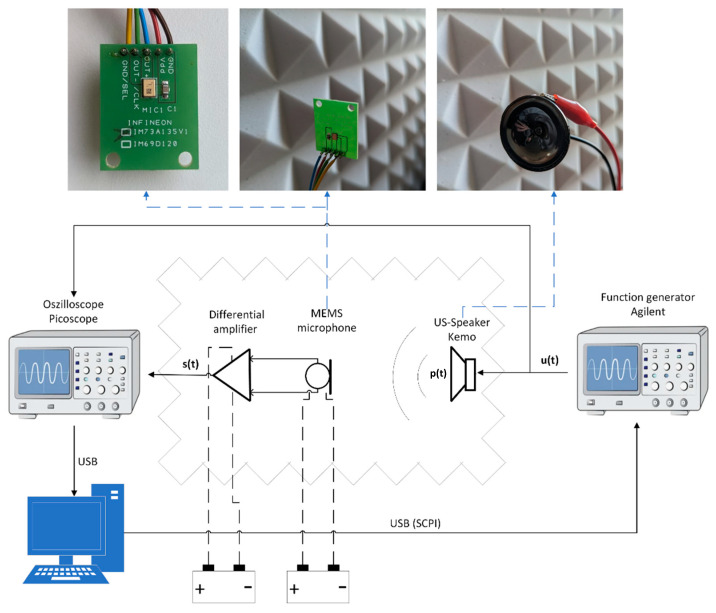
Block diagram of the experimental setup.

The transfer function and the SNR were measured with the experimental setup shown in Figure 1. Since ultrasonic loudspeakers are not standardized, a well characterized piezo loudspeaker was used (Kemo Electronic, L10, Geestland, Germany). $u\left(t\right)$ represents its voltage and $p\left(t\right)$ is the resulting pressure wave. $s\left(t\right)$ is the output signal of the MEMS microphone after differential amplification. The two channels $u\left(t\right)$ and $s\left(t\right)$ are recorded simultaneously by an oscilloscope (Pico Technology, Picoscope 544D, St. Neots, Cambridgeshire, UK). A function generator (Agilent, 32220a LXI, Santa Clara, CA, USA) was operated via SCPI by a PC to control the ultrasound speaker. The microphone was centrally positioned 19 cm in front of the speaker. The distance represents a compromise between high signal level and the fulfillment of the requirement for the far-field condition, where interference effects have a minor influence. In the sound field between the loudspeaker and the microphone, reflections were reduced with the aid of absorber material placed all around. The entire setup is controlled by a MatLab program running on the PC.

**Figure 2:**
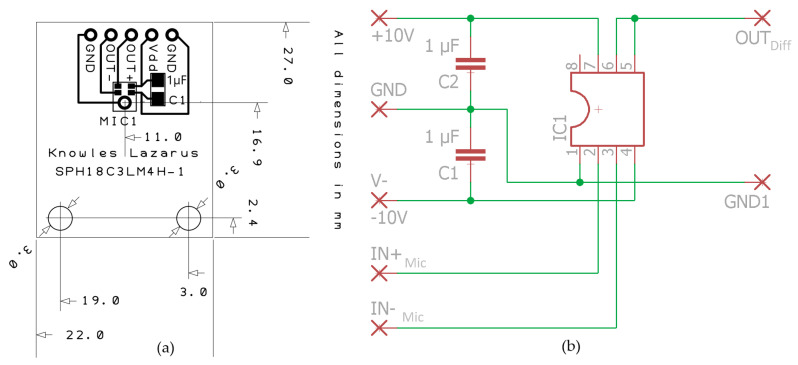
(**a**) Exemplary technical drawing of a microphone board; (**b**) electronic circuit for the differential amplifier.

To put the differential microphones into operation, the electronic circuit shown in Figure 2a was designed. It is based on a commercial differential amplifier (Burr-Brown, INA105, Tucson, AZ, USA). The differential amplifier has a gain of 1, therefore it has no effect on the sensitivity. In addition, it exhibits a low noise amplitude spectral density of 60 $\frac{\mathrm{nV}}{\sqrt{\mathrm{Hz}}}$, with 0.001% maximum nonlinearities and 0.01% maximum gain error [10]. The IN+/− inputs are suitable for the differential outputs of the different microphone boards. An exemplarily technical drawing is shown in Figure 2b. C1 represents a blocking capacitor. Through the openings at the bottom, the boards can be mounted in a soundproof way. The SMD components were soldered using a reflow oven (Lektor electronics: SMTprecision Lead-free Reflow Oven, SMTmax, Chino, CA, USA).

### 2.2. Methods

The SNR is determined using a traditional calculation according to Equation (1) [11]. This includes the power of the signal $P_{Signal}$ and the power of the noise floor $P_{Noise}$. Equivalently, the ratio of the effective values of the voltages, $U_{eff,Signal}$ and $U_{eff,Noise}$, can be used

(1) $$\mathrm{SNR}=10\log\frac{P_{Signal}}{P_{Noise}}=20\log\frac{U_{eff,Signal}}{U_{eff,Noise}}\;.$$

Another definition is often used for the case of an ultrasound field [12]. The power of a given band is calculated as the integral of the power spectral density (*PSD*)

(2) $${\mathrm{SNR}}_{US}=10\;\log\;\frac{\int_{f-\frac{1}{2\;}BW}^{f+\frac{1}{2}BW}PSD_{Signal}\left(f\right)}{\int_{f-\frac{1}{2\;}BW}^{f+\frac{1}{2}BW}PSD_{Noise}\left(f\right)}\;.$$

The determination of the transfer function was performed following the model of Novak [13], i.e., the deconvolution with an analytical exponential sweep $x\left(t\right)$ with the parameter L (refer to [13]) which depends on the time length of the sweep signal and the frequency range from $f_{1}$ to $f_{2}$ to be swept

(3) $$x\left(t\right)=\frac{f_{1}}{L}\exp\left(-\frac{t}{L}\;\right)\;u\left(-t\right)$$

In doing so, the sinusoidal frequencies contained at each point in time are convolved with the respective inverse group delay, which is why these components are transformed from the so-called deconvolution to a Dirac pulse. This is possible because the analytical signal $x\left(t\right)$ was calculated exactly for this purpose. However, if higher harmonics are contained in the signal, they occur with a lower group delay and thus in the deconvolved signal before the Dirac pulse [14].

In the time domain, the signal components are obtained as impulse responses $h_{m}$. In this way, the associated transfer function of the measured signal $s\left(t\right)$ is separated from its harmonics of orders m≥2 in time with $\Delta t_{n}$

(4) $$s\left(t\right)*x\left(t\right)=\sum_{m=1}^{\infty }h_{m}\left(t+\Delta t_{n}\right)=F^{-1}\left[F\left\{s\left(t\right)\right\}\cdot F\left\{x\left(t\right)\right\}\right]\;.$$

This calculation was performed analogously for the loopback channel $u\left(t\right)$ to confirm that a constant transfer function is obtained after deconvolution, as it should be according to theory, if the analytical signal was correctly inverted.

Using a Gaussian function, the impulse responses are then cut out and the transfer functions are obtained by Fourier transform. Thereupon, the transfer function of the recorded signal $F\left\{h_{1,\;s\left(t\right)}\left(\Delta t_{1}\right)\right\}$ is divided by one of the ultrasonic loudspeaker $H_{Speaker}$ which has been measured by Albuquerque et al. [15]

(5) $$H_{m}\left(f\right)=20\log\frac{F\left\{h_{m,\;\;s\left(t\right)}\left(\Delta t_{m}\right)\right\}}{H_{Speaker}\left(f\right)}\;.$$

## 3. Results

### 3.1. Transfer Function

**Figure 3:**
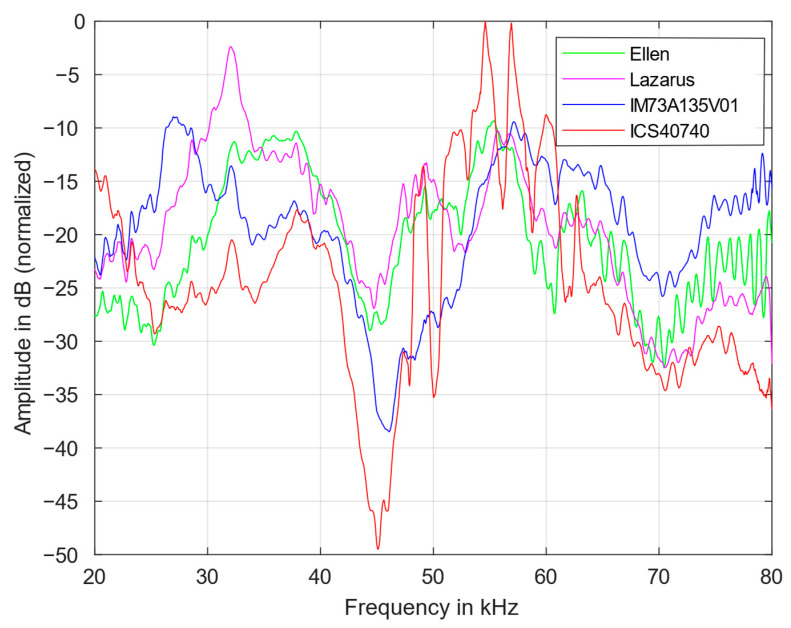
Fundamental transfer functions of the four MEMS microphones from 20 to 80 kHz.

Figure 3 shows the transfer functions of the four MEMS microphones between 20 and 80 kHz. The results were normalized according to the maximum. They are proportional to their respective detection sensitivities. It reveals that the sensitivity of a microphone is strongly frequency dependent. Above all, the frequency of the first mechanical resonance, which is formed primarily by the diaphragm mass, its stiffness, and the pre-volume as well as the sound channel [2], has an influence on the sensitivity. However, MEMS microphones often exhibit a second resonance significantly above 30 kHz, which is based on effects of the counter electrode and the back volume [16]. A sensitive microphone is particularly well suited for applications with low-level signals. Table 2 summarizes the frequencies with highest sensitivity measured in this work. The two peaks of the ICS40740 microphone originate from the same underlying resonance. Because of that, the average frequency of 55 kHz has been chosen.

For some models (IM73A135V01, Ellen and Lazarus), ultrasound sensitivities are specified by the manufacturer. The first resonance frequencies roughly agree with the values measured here. However, the manufacturer’s specifications are smoothed and provide considerably less information than the measurements shown here. Environmental conditions, such as temperature and relative humidity (values here: 19 °C, 50%, respectively), can lead to minor frequency shifts.

Above 70 kHz, the IM73A135V01 has the highest sensitivity, while between 30 and 40 kHz, the Lazarus and Ellen microphones stand out. The ICS40740 has a strongly fluctuating sensitivity in the range above 40 kHz and is therefore less suitable for ultrasonic applications.

**Figure 4:**
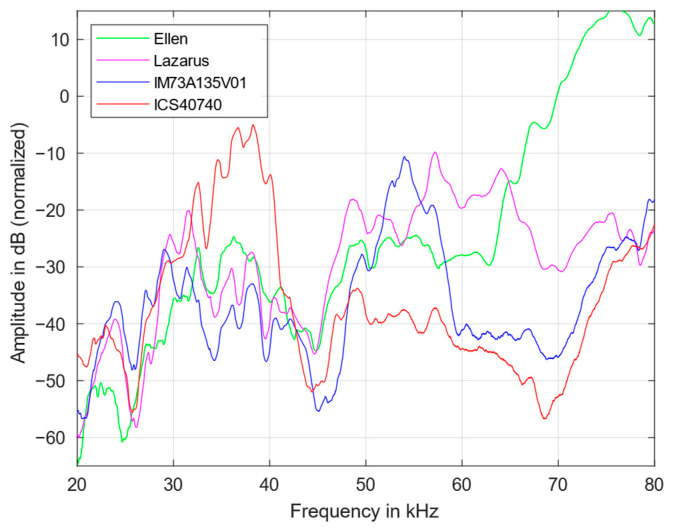
First harmonic transfer functions of the four MEMS microphones from 20 to 80 kHz.

**Figure 5:**
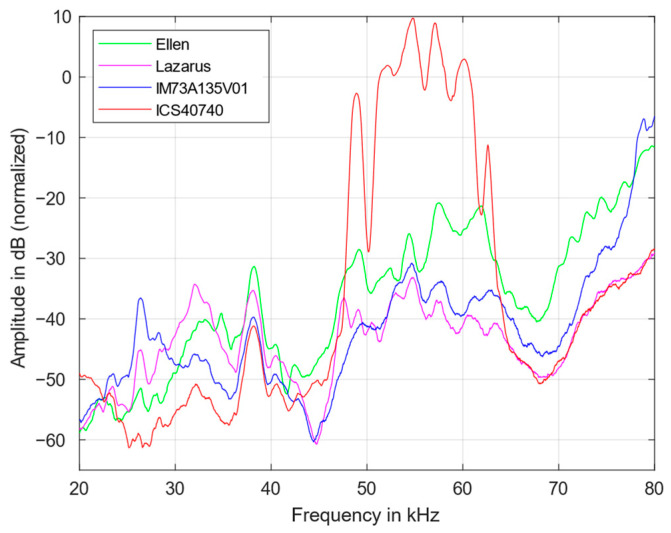
Second harmonic transfer functions of the four MEMS microphones from 20 to 80 kHz.

In order to estimate the proportion of harmonics, the transmission behavior of the first, see Figure 4, and second harmonic transfer function, see Figure 5, were measured. All harmonics were also normalized according to the maximum of the fundamental (in Figure 3). It was found that the contribution of the higher harmonics is also strongly frequency dependend.

Above 70 kHz, the harmonics are continously increasing for all microphones. The frequency dependency of the harmonics roughly follows the course of the fundamentals. However, the magnitude of the first harmonic is approximately 25 dB smaller than that of the fundamental transfer function, while from 20 to 40 kHz the second harmonic is smaller by another 10 dB on average. A large difference between the fundamental and the harmonics indicates a lower distortion factor and also reduces the SNR to a certain degree. Thus, the applicability of the microphones for low-level signals, as in photoacoustic measurements, improves.

In comparison, the IM73 exhibits a relatively low contribution of first and second harmonics. Above 70 kHz, the first harmonic of the Ellen microphone increases significantly; between 50 and 60 kHz, the contribution of the second harmonic is especially large for the ICS microphone and, at 55 and 57 kHz, has even a larger magnitude than the fundamental itself. This has also an influence on the SNR.

### 3.2. Signal-to-Noise Ratio

High microphone sensitivity is of little use if the SNR is low. For instance, in medical applications, moving the sensor or the patient as well as interferences can increase the noise significantly. In any case, it must be possible to clearly distinguish a low-level signal from the noise floor of the microphone. Therefore, in addition to a high sensitivity, a high SNR is desirable.

Due to its simplicity, the SNR was calculated according to the classical definition (Equation (1)). The SNR definition for the US range (Equation (2)) was also tested. It results in a value that is a few dB higher owing to a narrower bandwidth in the low-frequency range which is not relevant for a comparison of microphones. However, the calculation of the power density spectrum requires significantly more computing time.

**Figure 6:**
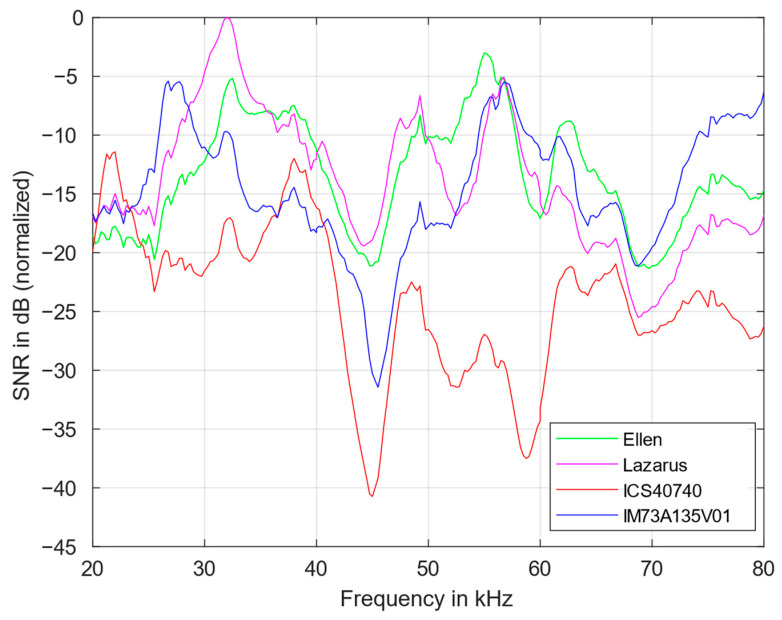
SNR of the four MEMS microphones from 20 to 80 kHz.

Figure 6 displays the SNR of the four MEMS microphones from 20 to 80 kHz which was normalized according to its maximum. It confirms a strong frequency dependency. The Lazarus and the Ellen microphone exhibit the highest SNR in the frequency range between 30 and 55 kHz. Above 65 kHz, the IM73 has an even better value; above 75 kHz it is more than 5 dB better than the next best. From 25 kHz on, the ICS40740 has the lowest SNR of all.

Good performances are possibly related to an optimization of stiffness, shape, and suspension of the membrane, as well as optimization of the front and back volumes and the adapted circuit elements of the ASIC in the microphone.

## 4. Conclusions

The performance of MEMS microphones in the near-ultrasonic range is strongly dependent on the microphone design and, as a result of that, on the frequency. This behavior is more pronounced in the ultrasonic than in the audible range. Therefore, an accurate characterization in the target frequency range is desirable before purchase. The results shown here provide an overview of the performance of various analog MEMS microphones in the near-ultrasonic range and are intended to help users select the most suitable microphone for their application.

Table 3 summarizes the SNR and the transfer function at different frequencies. The magnitude of the transfer function is proportional to the combined sensitivity of microphone and differential amplifier.

A high SNR is desirable for noisy applications. Table 3 clearly proves that a high sensitivity is not necessarily associated with a high SNR, which is essential for medical applications. A high SNR also allows the signal to be processed without many computationally expensive noise removal algorithms. If the sensor is to be miniaturized, it is helpful to dispense with other components, such as additional amplifiers. This is realistic if the selected microphone has a high sensitivity.

The authors divide the near-ultrasonic range into three sub-ranges and recommend the following microphones:

20–50 kHz: Lazarus (Knowles)

50–70 kHz: Ellen (Knowles)

>70 kHz: IM73A135V01 (Infineon)

To get precise values of the SNR for a certain sound field in the desired range, individual measurements with the respective setup are useful. Among others, the presented method of deconvolution for the relative course of the sensitivity can be used for this purpose, without expensive equipment such as sound calibrators.

## Figures and Tables

**Table 1:** Compared MEMS microphones and manufacturer characteristics for 1 kHz.

Manufacturer	Model	Sensitivity in dBV	SNR in dBV/Pa
Infineon	IM73A135V01	−38.0	73.0
TDK Invensense	ICS40740	−37.5	70.0
Knowles	Lazarus PH18C3LM4H-1	−38.0	68.5
Knowles	Ellen SPW0878LR5H-1	−38.0	65.0

**Table 2:** Resonance frequencies of the four MEMS microphones.

Microphone	Frequency in kHz	
	First Resonance	Higher Resonances
IM73A135V01	28	40/57
ICS40740	20	40/55
Lazarus	33	48/55
Ellen	37	48/55

**Table 3:** Transfer function (H) and SNR of the four microphones at different frequencies.

Microphone	20 kHz		40 kHz		75 kHz	
	SNR	H	SNR	H	SNR	H
IM73A135V01	−16.34	−25.35	−17.94	−23.31	−10.16	−19.97
ICS40740	−19.88	−17.12	−16.14	−24.28	−24.64	−32.72
Ellen	−25.72	−30.94	−15.14	−19.33	−15.03	−26.67
Lazarus	−16.90	−26.50	−12.03	−18.53	−18.47	−29.40

## Data Availability

The measurement data will be provided by the authors upon request. Contact the correspondence address.
